# A wearable device for at-home obstructive sleep apnea assessment: State-of-the-art and research challenges

**DOI:** 10.3389/fneur.2023.1123227

**Published:** 2023-02-07

**Authors:** Ngoc Thai Tran, Huu Nam Tran, Anh Tuan Mai

**Affiliations:** Faculty of Electronics and Telecommunication, VNU University of Engineering and Technology, Hanoi, Vietnam

**Keywords:** OSA, SCOPER, machine learning, wearable device, COVID-19

## Abstract

In the last 3 years, almost all medical resources have been reserved for the screening and treatment of patients with coronavirus disease (COVID-19). Due to a shortage of medical staff and equipment, diagnosing sleep disorders, such as obstructive sleep apnea (OSA), has become more difficult than ever. In addition to being diagnosed using polysomnography at a hospital, people seem to pay more attention to alternative at-home OSA detection solutions. This study aims to review state-of-the-art assessment techniques for out-of-center detection of the main characteristics of OSA, such as sleep, cardiovascular function, oxygen balance and consumption, sleep position, breathing effort, respiratory function, and audio, as well as recent progress in the implementation of data acquisition and processing and machine learning techniques that support early detection of severe OSA levels.

## 1. Introduction

Obstructive sleep apnea (OSA) is a common sleep disorder characterized by repeated interrupted upper airflow during sleep. Previous studies have reported that sleep disorders, such as OSA, are associated with heart disease (1), diabetes type 2 (2, 3), stroke (4, 5), and depression (6). Sleep apnea affects the quality of life and working performance and is associated with other diseases; however, it does not lead to death by stopping breathing. This may be why OSA is underestimated as not many people are aware of or diagnosed with it.

The prevalence of OSA has been rising and affecting all countries. An investigation by Benjafield AV presents a global prevalence and burden that OSA may cause (using AHI and AASM 2012 criteria), in which “936 million [95% confidence interval (CI) 903–970] adults aged 30–69 years (men and women) may have mild to severe OSA and 425 million (95% CI 399–450) adults aged 30–69 years may have moderate to severe OSA globally” (7). In Vietnam, ~8.5% of adults, which is equivalent to ~5.9 million persons, have an AHI of >15 (8), especially those with systemic hypertension and chronic obstructive pulmonary disease (9, 10).

**Figure 1:**
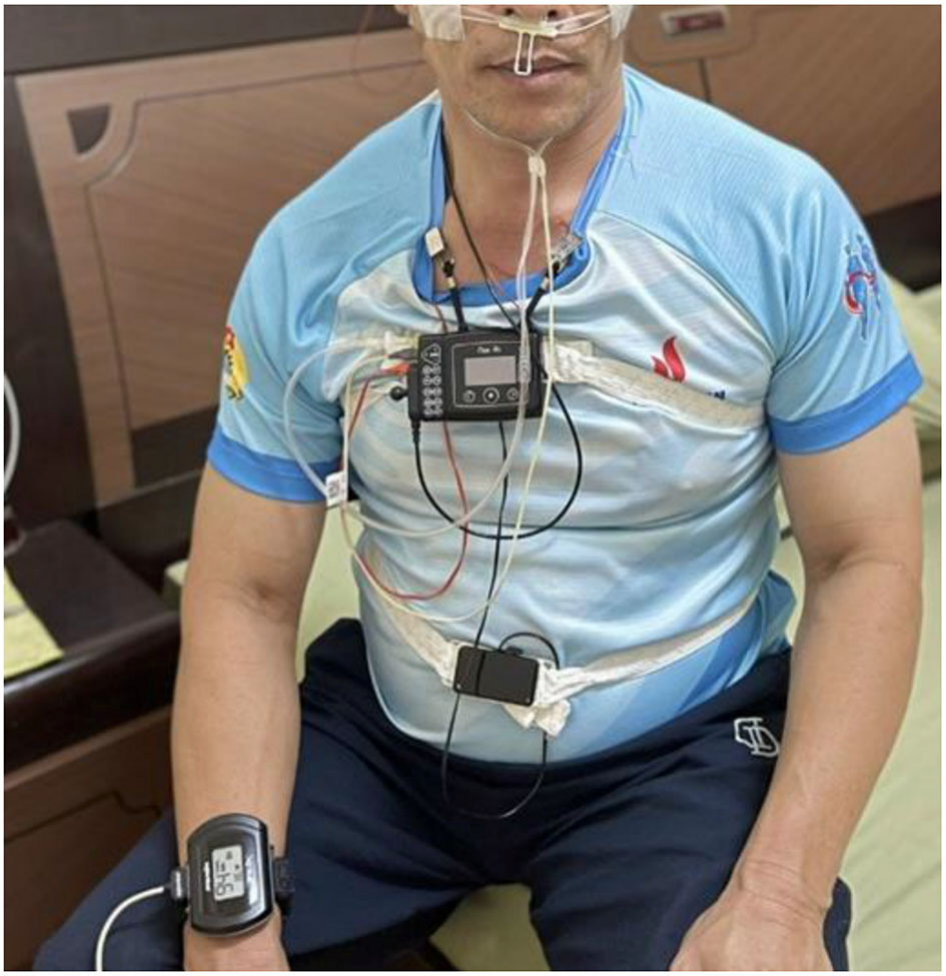
OSA patient is diagnosed using a multi-electrode PSG at a sleep laboratory.

Despite the negative impact of OSA on health and its increasing prevalence, poor awareness about OSA has been reported in many countries, such as Singapore (11), Saudi Arabia (12), Pakistan (13), and, unfortunately, even worse in mid-and low-income countries. Currently, polysomnography (PSG) is the gold standard for evaluating sleep apnea/hypopnea (14). PSG can record multiple parameters of a person, such as brain waves [electroencephalography (EEG)], nasal–oral airflow, thoracoabdominal effort, and snoring, within the 8 h sleeping at a hospital (Figure 1).

During the COVID-19 pandemic, almost all resources were reserved for testing, diagnosing, and treating patients with COVID. Hospitals did not have sufficient facilities or human resources for other diseases, even in emergencies. This has led to more people with OSA not being screened and diagnosed, resulting in a long waiting queue of people who wish to meet the physician for a diagnosis at the laboratory. During the post-COVID period, countries prioritized some urgent diseases, such as cardiovascular diseases, cancers, hepatitis B, and dengue fever, which increased the number of OSA cases and the severity. This may be why people seem to be paying more attention to alternative home OSA detection solutions. Therefore, early diagnosis of OSA and suitable proposed therapies for patients have interested many companies.

This study aims to review the possible impact of the SARS-CoV-2 virus and OSA and summarize state-of-the-art assessment techniques for out-of-center detection of the main characteristics of OSA, such as sleep, cardiovascular function, oxygen balance and consumption, sleep position, breathing effort, respiratory function, and audio. The study also aims to review recent progress in the implementation of data acquisition and processing and machine learning techniques that support the early detection of severe OSA levels.

## 2. Association between COVID-19 and OSA

The SARS-CoV-2 virus causes the infectious COVID-19, which affects the respiratory system. It can cause lung complications; therefore, it affects the airflow of patients, particularly those with OSA which is usually characterized by upper airway obstruction at night.

Maas et al. evaluated 5,544,884 patient records, of which 9,405 were COVID-19-infected cases, to identify possible links between OSA and the risk of COVID-19 infection and the severity of the disease. The study found that among patients with COVID-19, ~34% were hospitalized and 19% were diagnosed with respiratory failure. The prevalence of hospitalized patients with OSA was higher than that of those without OSA [15.3 vs. 3.4%, *p* < 0.0001; odds ratio (OR) 5.20, 95% CI (4.43, 6.12)]. A similar result was found regarding respiratory failure rate [OR, 1.98; 95% CI (1.65, 2.37)] (15).

Labarca et al. conducted a study of COVID-19-infected people (≥18 years of age) to determine the association of OSA with long-term symptoms and inflammatory cytokines (4 and 12 months after the COVID treatment). The OSA group demonstrated poor effects on insulin resistance levels, metabolic change, cytokine levels, and symptoms compared with the non-OSA group (16).

A study by Alemohammad et al. in 2021, conducted with 275 (adult) participants diagnosed with OSA, reported that pro-inflammatory characteristics of OSA may increase the risk of COVID-19, and severe OSA was associated with higher COVID-19 prevalence among patients with OSA (17).

The prevalence of SARS-CoV-2, being a respiratory disease virus, is thought to be associated with comorbid conditions, including age, male sex, hypertension, elevated body mass index/obesity, diabetes, and chronic obstructive lung disease. In addition, previous studies found that OSA was independently associated with an increased risk of developing severe COVID-19; however, OSA may be a risk factor for severe COVID-19 (18–20).

COVID-19 has not yet been completely controlled because it will take years for humans to fully understand and synthesize specific drugs for such a disease. Furthermore, much work should be done to improve OSA screening and achieve more effective treatment.

## 3. Detection of main characteristics of OSA

The early diagnosis of OSA and the development of suitable therapies for patients have interested many companies. To date, PSG has been the gold standard for evaluating sleep apnea/hypopnea (14). PSG can record multiple parameters of a person, such as brain waves, nasal-oral airflow, thoracoabdominal effort, and snoring, within at least 8 h sleeping in a sleep laboratory. With the continuous support of a technician, sleep is continuously recorded, and other sleep conditions may be observed. However, the PSG test requires a person to go to a sleep laboratory and stay overnight for an entire test, which makes one feel uncomfortable and inconvenient. In addition, costly PSG will limit the number of patients tested, especially those in mid- and low-income countries. Therefore, the development of alternatives, such as out-of-center sleep tests, allowing a person to evaluate and predict a possible OSA at home [Home Sleep Testing (HST)] plays an important role and will help more people access such healthcare solutions and services.

Sleep studies in engineering, especially developing portable or wearable mobile devices for evaluating OSA at home, have received considerable attention from research groups worldwide. The studies in the literature focused on six main topics summarized as SCOPER categorization (21): Sleep; Cardiovascular; Oximetry; Position; Effort; and Respiratory.

HST is an attractive alternative to PSG for insurance companies and patients because it is affordable. For a patient, having a sleep test at home is much more convenient than spending a whole night in a remote room and being monitored during sleep at a laboratory. In addition, with HST, one does not need to book a bed in the presence of a sleep technologist to monitor the sleep parameters.

This potential market for commercial HST devices has attracted many investors (Table 1). The HST devices are normal full-fill type II, III, and IV levels of sleep study, among which type II approaches full PSG measurements outside the laboratory. The main difference from type 1 devices is that the sleep study is performed without a medical technician. The HST device usually is built-in with an internal memory that allows 300 h data storage with a disposable or rechargeable battery. The weight of the HST device (including the battery) does not exceed 350 g. In addition, the user (patient or medical doctor) can refer to the test results at home (Table 1).

**Table 1:** Commercial HST devices.

**Device**	**Company**	**Electrode**	**Data storage (GB)**	**Data recording time (hours)**	**Reference**
Nox T3s	Nox Medical	Breathing effort, respiratory sound, gravity, position, flow, snore, PWA; pulse, SpO2; heart rate	4 built-in	24 with 1 AA battery	noxmedical.com
ApneaTrak Type 3 HSAT	Cadwell Industries Inc.	Thoracic/abdominal effort, snore sensor, thermistor, SpO2, pulse rate, body position.	0.1 built-in	18	cadwell.com/apneatrak
BWMini HST Compass	Neurovirtual USA Inc.	Effort, pressure transducer, SpO2, pulse, plethysmography, body position, luminosity sensor	3.2 built-in	12 with 1 AA battery	neurovirtual.com
Zmachine Synergy	General Sleep Corporation	EEG, respiratory effort (RIP), snore, SpO2, pulse rate, body position	8.0 built-in	300 with Lithium Ion 3.7 VDC	generalsleep.com
Somté	Compumedics USA	Nasal pressure, snoring, thoracic and abdominal effort, body position, SpO2, pulse rate, limb movement, EEG, EOG, EMG, ECG	Inserted card	36	compumedics.com
MediByte Jr	BRAEBON Medical	Airflow, snore, SpO2, pulse rate, chest effort, body position, CPAP pressure, PPG	0.2 built-in	18 with 1 AA battery	www2.braebon.com
Alice NightOne	Philips	Flow, snoring, thoracic effort, SpO2, heart rate, body position, plethysmogram.	0.4 built-in	10 with 1 AA battery	usa.philips.com
Cerebra Sleep System	Cerebra	Brain waves (EEG), Eye movements (EOG), Respiratory data, Pulse oximetry, Heart rate (ECG), Chin and leg activity (EMG), Body position, Snoring, Oxygen saturation (SpO2).	0.3 built-in	13 with 1 AA battery	cerebra.health

In the research and development phase, many research groups study SCOPER parameters to introduce more products to manufacturers, focusing on reducing the number of sensors and improving measurement accuracy.

Sleep parameters can be studied using electrooculography (EOG), EEG, and electromyography (EMG) to acquire sleep data. The sensors can be placed on the scalp, the forehead, the ear, or the chest (22–25).

Shustak et al. proposed an ambitious project for an in-home OSA detection system using temporary-tattoo EEG, EOG, and EMG electrode arrays (26). They demonstrated that a polyurethane film-based electrode array allowed for the simultaneous monitoring of EMG, EOG, and EEG signals during napping and night sleep. The electrode array was interfaced with a built-in low-energy Bluetooth and an Intan SoC PCB for data collection and processing.

A simple configuration of microphones (Earthworks M23, Behringer ECM8000) was used to record snoring sounds during sleep using a combination of a convolutional neural network (CNN) and a recurrent neural network (RNN), as reported by Xi Long et al. Overall, accuracy of 95.3 ± 0.5%, sensitivity of 92.2 ± 0.9%, and specificity of 97.7 ± 0.4% were obtained in the study (27).

OSA can be studied by combining multiple approaches, such as an electrocardiogram (ECG), EEG, and sPO2. Each approach demonstrated the possibility of developing advanced devices for OSA detection.

## 4. Data acquisition and processing

**Figure 2:**
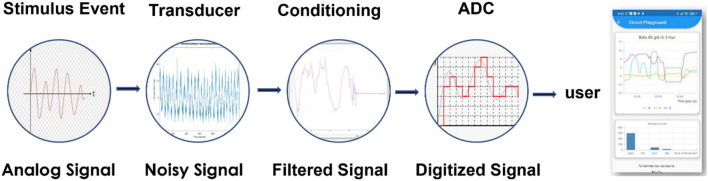
A signal is detected by a transducer and converted into digital form, processed, and displayed on a user interface.

In general, a non-electric stimulus signal is converted into an electrical signal by a transducer in analogic form, which is then transformed into digital form using an ADC and finally to analogic form again, enabling the user to see the result of the measurement (Figure 2). However, the input data are disturbed by existing noise in the measuring environment.

The signal-to-noise ratio can be improved using both hardware and software. For example, in a sleep apnea study, one should select the frequency range in which the data will be collected, and naturally, the out-of-range ones are filtered out. As a result, the developer will probably define the specific characteristics of the selected sensors for specific purposes (Table 2). Furthermore, selecting suitable sensors characterized within a limited time will also be effective for the calibration step.

**Table 2:** Frequency range and selected sensors used in sleep apnea.

**Sensor**	**Measurement**	**Range**	**Reference**
Accelerator	Wake and sleep periods	0.1 Hz−0.4 Hz	(28)
Acoustic sensor	Respiratory sounds	100–1,400 Hz	(29)
Acoustic sensor	Tracheal sound	100–3,000 Hz	(30)
Acoustic sensor	Sound transmission in respiratory system	150–500 Hz	(31)
Pressure sensor	Pressure	0.06–1.7 Hz	(32)
Captive microphone	Respiratory sound	30–126 Hz	(33)
Captive microphone	Respiratory sound	20–6,000 Hz	(34)
Acceleration sensor	Actigraphy, body position	1–4 Hz	(35)

In addition to the hardware approach, the Fast Fourier Transform (FFT), a means of mapping a signal, either in the time or space domain, to its spectrum in the frequency domain, is usually utilized in sleep apnea studies. FFT allows the computation of discrete Fourier transform (DFT) of a sequence with high efficiency. Therefore, the system may require less performance hardware and significantly reduce computation time. For example, Belhaouari et al. implemented an ECG using FFT to diagnose sleep apnea with 100% accuracy (36). In addition, overnight breath recording data were collected, conditioned by the FFT, and learned using random forest (RF) and support vector machine (SVM), which offer an overall accuracy of >90% (37).

The sleep stage was evaluated using brainwave signals from EEG and FFT to improve accuracy (~96.54%), and the performance of automated sleep classification was reported by Delimayanti et al. (38). FFT can also monitor non-invasive respiration using ECG-derived respiratory (EDR) signals. A combination of FFT preprocessing, linear and quadratic Discriminant (LD and QD) models, K-nearest neighbor classifiers (KNN), SVM, and an artificial neural network (ANN) offered an evaluation with 100% accuracy (39).

**Figure 3:**
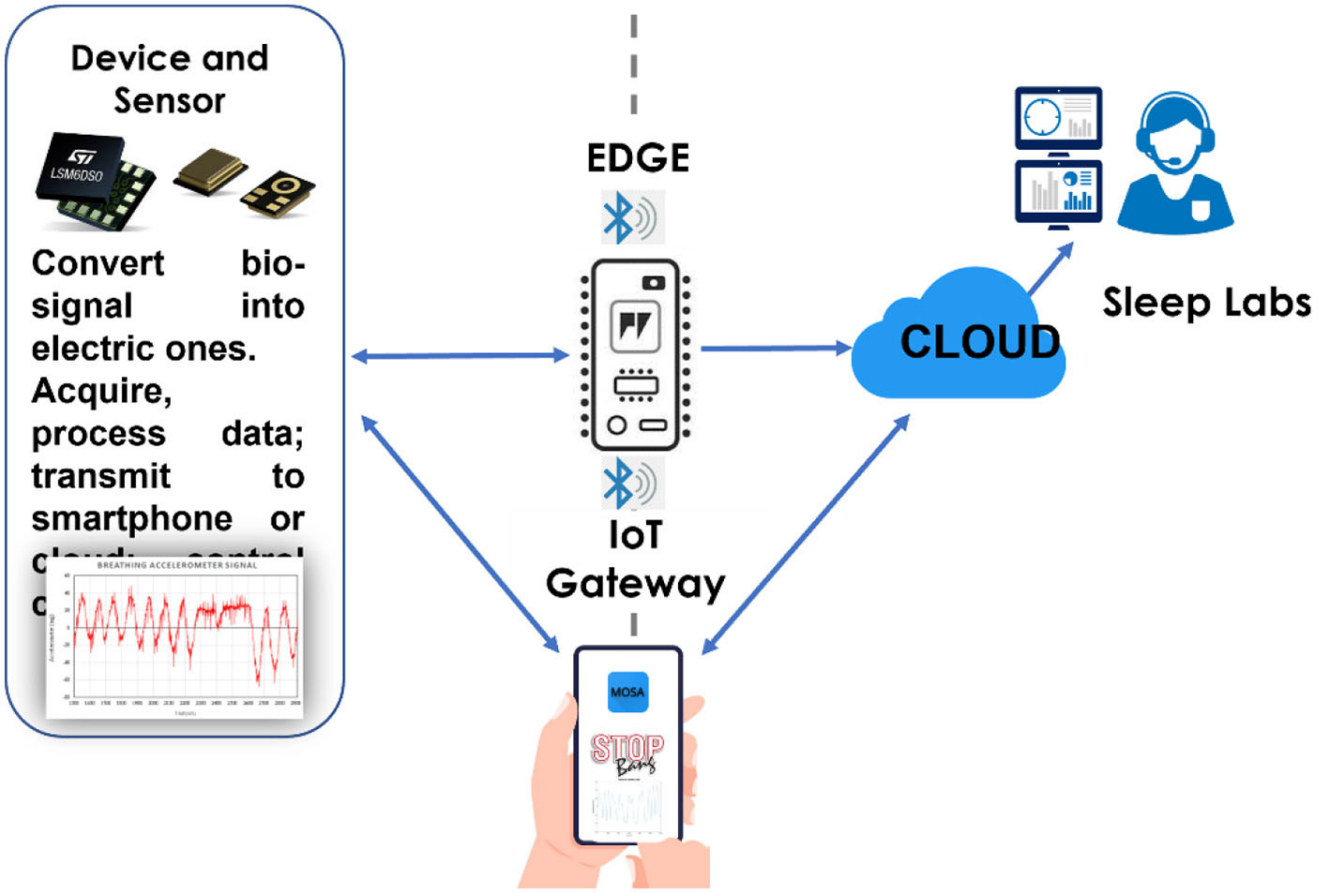
The data can be deposited on a service cloud.

The acquired and processed data can be deposited on the cloud using various options. For example, the developer can either select private or public, free or paid, or hybrid clouds from different providers such as Amazon, Google, Microsoft, Alibaba, Oracle, IBM, Digital Ocean, and Dropbox (Figure 3).

## 5. Machine learning in OSA study

The wearable device has fewer sensors for at-home sleep apnea detection than the HST (commercially available). In addition, machine learning and artificial intelligence (AI) are used after data processing to improve the accuracy and precision of the measurement.

RF, SVM, Logistics Progression, and Naive Bayes classifier are the models typically used in sleep studies. Each model may be suitable for one or two different signal types. For example, regularized logistic regression (LR) appears faster for tracheal breathing sounds than RF. In contrast, RF seems better than LR in blind-testing accuracy, specificity, and sensitivity; therefore, both are good for OSA research (40).

Álvarez et al. conducted research with 303 patients with OSA on adult sleep apnea screening for >2 years at home using airflow and SpO2 sensors. The study implemented SVM as a machine-learning model and showed that the accuracy was >95% using both SpO2 and airflow data (41).

Usually, a sleep study involves more than one machine learning model to improve accuracy, specificity, and sensitivity. For example, Gallo et al. implemented ML, KNN, RT, SVM-R, LR, and Adaboost in their research to determine the optimal one in an OSA study (42). In the study by Wu et al., RF, KNN, and SVM classifiers were used to classify sleep apnea using EEG signals with an average accuracy rate of 88.99% after 10-fold cross-validation (43).

Research groups have implemented deep learning in OSA studies. Deep learning is a particular type of machine learning that can handle different types of data, such as images, videos, and raw data. Deep learning processes a large dataset. This requires more computing power than human intervention.

Yue et al. reported the multi-resolution residual network (Mr-ResNet) detecting nasal pressure airflow signals from a PGS at sleep laboratories. According to the authors, the Spearman correlation for AHI between the obstructive sleep apnea smart system (proposed by the research group) and the registered polysomnographic technologist score was 0.94 (*p* < 0.001) and 0.96 (*p* < 0.001), respectively. Furthermore, Cohen's kappa scores for classification obtained by the two technologists were 0.81 and 0.84, respectively (44).

The 1D CNN model was used in the study by Lin et al. to develop a sleep apnea system. The proposed model was composed of 10 identical CNN-based feature extraction layers, one flattened layer, four identical classification layers, and a softmax classification layer. The ECG data were extracted from two sleep laboratories to train the model. For the per-recording classification, the accuracy was 97.1%, specificity was 100%, and sensitivity was 95.7% (45).

Recent research reported by Nguyen et al. demonstrated a head-based sleep-aid system for promoting fast falling into sleep and improving the accuracy of sleep tracking. Using a multi-sensor configuration (accelerator, PPG sensor, and bioelectrodes) for EEG, EOG, sleep posture, breathing, and heart rate, the author implemented a set of algorithms to achieve great agreement with results obtained by a PSG (46).

## 6. Discussion

Home OSA assessment has many advantages; however, some challenges must be overcome to make such alternative solutions more popular and accepted by patients, physicians, and medical insurance companies.

Existing out-of-center OSA assessment devices employ fewer components than a PSG; however, they still have complicated moving parts that may not be convenient for patients with backgrounds such as chronic obstructive pulmonary disease, congestive heart failure, and neuromuscular diseases. In addition, to our knowledge, the average price of an HST device is >2,500 USD. However, a patient can sometimes hire an HST device for ~300 USD per night.

### 6.1. Challenges for HST development

HST, as discussed earlier, has many advantages for patients; however, there are challenges that solution and product developers must overcome to make such testing methods more prevalent for patients, physicians, and medical insurance companies. A review of the literature reveals the following key challenges for device developers.

#### 6.1.1. Accuracy improvement

HST uses less hardware on board; therefore, assessments may miss important information about OSA. HST does not record sleep, but only breathing and/or a stop in breathing; therefore, the accuracy needs to be improved by combining two or three sensors on a board. Accuracy can be improved by implementing suitable machines and deep learning techniques.

#### 6.1.2. Limit of the tested population

Existing HST devices employ fewer components than PSG; however, they still have complicated moving parts such as pumps, electrodes, and control panels. Such a configuration may not be suitable for patients with chronic obstructive pulmonary disease, congestive heart failure, or neuromuscular disease. For the patient's convenience, the system configuration must be simpler and more comfortable. Two or three types of sensors on a small board may be sufficient to collect two or three types of signals associated with OSA characteristics.

#### 6.1.3. Collaboration with physicians

To date, most studies on system development, including devices and software, for OSA detection and diagnosis have been reported by technical teams; however, technology and solutions must be reviewed and revised by physicians from sleep laboratories. A loose collaboration with physicians may lead to the number of samples (tested participants) not being large enough for a sustainable evaluation of the device and solution. The strong support of physicians will guide the engineering team in the right and shortest path for medical equipment that patients will accept. In addition, this support can help increase the number of tested participants according to different variants (age, sex, or medical history compatible with existing solutions and services) to improve the accuracy of the algorithms and machine learning techniques.

### 6.2. The trend in OSA device development

The trend in developing mobile devices for OSA detection is a smart combination of a simple hardware configuration that integrates two or three kinds of sensors on a small PCB. Suitable data acquisition and processing should be applied to remove artifacts and enhance the signal-to-noise ratio. Furthermore, the data of a study should be sufficiently large to implement machine or deep learning models and to enhance the accuracy, sensitivity, specificity, and precision. Importantly, data mining should be conducted in a case–control study using the PSG method.

Data processing and storage appear to benefit from the development of the information and electronics industries. Advanced battery technology allows a wearable sleep-testing device to operate for 10 h or more with a compact rechargeable lithium-ion polymer battery. Developers can also choose wire or wireless charging modes for their solution. The cutting-edge technology with Bluetooth Ultra Low Energy and nano-range energy consumption integrated circuits and sensors make it possible for the device to work a whole night without recharging. Machine learning models can run on a Chip, Edge Device, or Service Cloud.

## 7. Conclusion

Today, digital transformation in healthcare is taking advantage of cutting-edge technologies and innovations to deliver sustainable service and medical solutions to patients, medical staff, and healthcare bodies. Home sleep apnea test (HSAT) is a promising and alternative sleep study solution for people with OSA that may help to save time and money while enabling improved interaction between physicians and patients. HSAT involves almost all major digital transformation trends in healthcare including health wearables, AI screening, disease history analysis, e-doctor, and data aggregation. However, to popularize such an advanced solution to one-seventh of the world's adult population (~1 billion people, especially people in mid- and low-income countries), great efforts of the developers, medical staff, patients should be made to simplify the hardware with a smaller number of sensors, to improve the accuracy of the test, to use with ease, and to reduce the service cost of the solution.

## Author contributions

AM: conceptualized, validated, and wrote the original draft. AM, NT, and HT: methodology and writing review. All authors have contributed to the manuscript and approved the submitted version.
